# High frequency of loss of PTEN expression in human solid salivary adenoid cystic carcinoma and its implication for targeted therapy

**DOI:** 10.18632/oncotarget.3411

**Published:** 2015-03-20

**Authors:** Han Liu, Li Du, Ru Wang, Chao Wei, Bo Liu, Lei Zhu, Pixu Liu, Qiang Liu, Jiang Li, Shi-Long Lu, Jing Xiao

**Affiliations:** ^1^ Department of Oral Pathology, College of Stomatology, Dalian Medical University, Dalian, China; ^2^ Department of Otolaryngology, University of Colorado School of Medicine, Aurora, CO, USA; ^3^ Department of Stomatology, First Affiliated Hospital, Dalian Medical University, Dalian, China; ^4^ Institute of Cancer Stem Cell, Dalian Medical University, Dalian, China; ^5^ Department of Oral Pathology, 9^th^ People's Hospital, School of Medicine, Shanghai Jiao Tong University, Shanghai Key Laboratory of Stomatology, Shanghai, China

**Keywords:** salivary gland tumors, adenoid cystic carcinoma, PTEN, targeted therapy

## Abstract

Salivary gland tumor (SGT) is one of the least studied cancers due to its rarity and heterogeneous histological types. Here, we reported that loss of PTEN expression was most frequently found in the poorly differentiated, high grade solid adenoid cystic carcinomas. Loss of PTEN expression correlated with activation of mTOR by increased phosphorylated S6 ribosome protein. We further functionally studied the role of PTEN in a pair of human SACC cell lines, SACC-83 and SACC-LM. Reduced PTEN level was correlated with the metastasis potential. When we knocked down PTEN in the SACC-83 cell line, we observed increased proliferation and enhanced migration/invasion *in vitro*, and increased tumor size *in vivo*. We further tested the therapeutical effect by applying a PI3K/mTOR inhibitor NVP-BEZ235 to both SACC cell lines. Decreased cell proliferation, increased apoptosis, as well as reduced cell migration/invasion were observed in both cell lines upon the NVP-BEZ235 treatment. Moreover, the NVP-BEZ235 treatment in a SGT xenograft mouse model significantly reduced primary tumor size and lung metastasis. Taken together, our results demonstrated that PTEN is a potent tumor suppressor in human SGTs, and targeting PI3K/mTOR pathway may be effective in the targeted therapy for human SGT patients with loss of PTEN expression.

## INTRODUCTION

Salivary gland tumors (SGTs) are uncommon tumors, which account for approximately 3–6% of all head and neck tumors. The annual incidence is approximately about 2.5 cases per 100, 000 people in U.S. and between 4 and < 0.5 cases per 100, 000 people worldwide [1, 2]. SGTs occur either in major salivary glands including parotid, submandibular, and sublingual glands or in minor glands. SGT is one of the most heterogeneous tumors, which is composed of more than 20 histopathological subtypes with widely varied clinical outcomes [3, 4]. Among these subtypes, salivary adenoid cystic carcinoma (SACC) and mucoepidermoid carcinoma (MEC) are two most common cancers in human SGT patients. Although radiation or occupational exposure may play roles in SGT tumorigenesis, the etiological factors for SGTs are still largely unknown [1–4]. Molecular events associated with human SGTs are poorly understood although chromosome translocation and gene fusions are commonly seen in both SACC and MEC [5]. Both rarity of incidence and heterogeneity of pathology pose challenges for SGT study, making it one of the least studied tumor type [6]. Because of these circumstances, effective treatments for these tumors are very limited. There is an urgent need to understand the molecular mechanisms of formation and malignant progression in SGTs, which will be translated into novel therapeutic approaches to improve the current treatment for SGT patients.

Phosphatase and tensin homologue deleted on chromosome 10 (PTEN), best known as a lipid phosphatase, negatively regulates cell proliferation and survival through antagonizing the phosphatidylinositol 3-kinase (PI3K) signaling [7]. Somatic mutations, deletions and epigenetic silencing of PTEN have been reported in a variety of solid tumors, making PTEN one of the most important tumor suppressors in human cancers [8]. Loss of functional PTEN leads to constitutively activate downstream components of PI3K pathway, such as AKT and mTOR, providing an opportunity for targeted therapy using PI3K/AKT/mTOR inhibitors [7, 8]. In human SGTs, although there were sporadically reports of PTEN alterations [9–11], it is still unclear the role of PTEN in SGT tumorigenesis. Furthermore, it is inconclusive if targeting PI3K/AKT/mTOR pathway would be effective for the treatment of human SGTs.

To address these questions, we first performed a detailed immunohistochemistry analysis of PTEN in multiple types of human SGTs, and normal salivary gland tissues. We then knocked down PTEN to assess its role in human SGTs both *in vitro* and *in vivo*. Finally, we tested a small molecule inhibitor targeting PI3K/mTOR both *in vitro* and in a xenograft SGT mouse model to evaluate its therapeutic effects.

## RESULTS

### PTEN is ubiquitously expressed in human normal salivary glands

**Figure 1:**
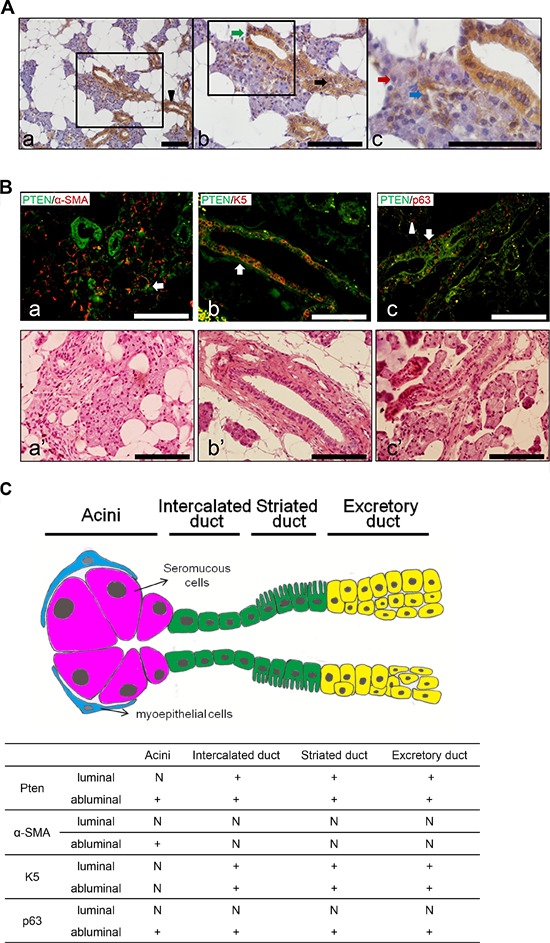
PTEN, α-SMA, K5 and p63 distributions in human normal salivary glands **A.** Immunohistochemistry (IHC) staining of PTEN in human normal parotid glands. Note that PTEN showed strong expression in the excretory duct (a, black arrowhead), intercalated duct (b, black arrow), striated duct (b, green arrow) cells and abluminal (myoepithelial) cells of serous acini (c, blue arrow), but not in luminal (epithelial) cells of serous acini (c, red arrow). Scale bar: 100 μm. **B.** Double immunofluorescence (IF) staining of PTEN (a-c, green) with α-SMA (a, red), K5 (b, red) and p63 (c, red) in human normal salivary glands. α-SMA was co-localized with PTEN in the abluminal cells of acini (white arrow in a); PTEN and K5 double positive staining was shown in abluminal cells of ducts (white arrow in b); PTEN and p63 are double-positive in abluminal cells of acini (white arrowhead in c) and ducts (white arrow in c); The corresponding HE slides were listed below (a'–c') for better visualization. Scale bar: 100 μm. **C.** Summary of PTEN, α-SMA, K5 and p63 expression pattern in human normal salivary gland. +: > 90% of positive cells, N: negative.

To investigate the role of PTEN in human salivary gland tumors, we first examine the expression pattern of PTEN in normal human salivary glands. As shown in Figure 1A, a strong cytoplasmic expression of PTEN was observed in both luminal (epithelial) and abluminal (basal) cells of intercalated ducts, striated ducts and excretory ducts of normal human parotid glands. PTEN is also consistently expressed in the abluminal (myoepithelial) cells, but barely expressed in the luminal (epithelial) cells of acini. To further examine the distribution of PTEN with other salivary gland markers, we performed double IF of myoepithelial cell marker α-smooth muscle actin (α-SMA), basal cell marker Keratin 5 (K5), and abluminal (both myoepithelial and basal cells) cell marker p63. As shown in Figure 1B, PTEN is co-localized with α-SMA in the abluminal (myoepithelial) cells, and both are not expressed in the luminal cells of acini. PTEN is also co-expressed with K5 in the abluminal cells of ducts. Furthermore, PTEN and p63 are double-positive in abluminal cells of acini and ducts, including both myepithelial cells and basal cells. The distribution of PTEN, α-SMA, K5 and p63 expression pattern in normal human salivary gland is summarized in Figure 1C.

### Loss of PTEN expression was predominantly found in human solid salivary adenoid cystic carcinomas

**Figure 2:**
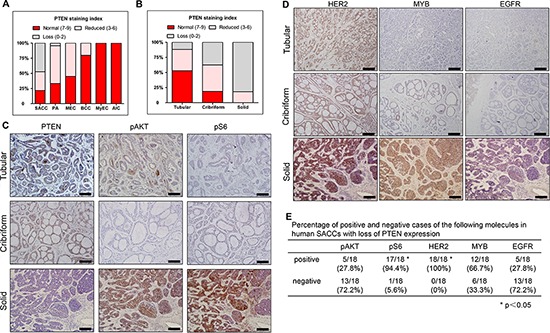
PTEN expression and its correlation with pAKT, pS6, HER2, MYB and EGFR in human salivary gland tumors **A.** Quantification results of PTEN IHC in human salivary gland tumors. SACC: salivary adenoid cystic carcinomas, PA: pleomorphic adenomas, MEC: mucoepidermoid carcinoma BCC: basal cell carcinoma, MyEC: myoepithelial carcinoma, (BCCs), AIC: acinic cell carcinoma. **B.** Quantification results of PTEN IHC in three patterns of human SACC. **C.** Representative images of PTEN, pAKT and pS6 IHC staining in three patterns of human SACCs. The scale bars represent 100 μm. **D.** Representative images of IHC of HER2, MYB and EGFR in three patterns of human SACCs. The scale bar represents 100 μm. **E.** Percentage of positive and negative cases of pAKT, pS6, HER2, MYB and EGFR in human SACCs with loss of PTEN expression. Positive of these molecules were defined as the staining index ≥ 3. Loss of PTEN expression was defined as the staining index ≤ 2.

We then examined PTEN expression by immunohistochemistry (IHC) in a total of 114 human salivary gland tumors (SGTs). Results of PTEN IHC staining were summarized in Figure 2A, 2B, and Table 1. The average PTEN staining index is 5.55 (Table 1). Loss of PTEN expression (defined as PTEN staining index ≤ 2) was identified in a total of 23.7% (27/114) of all SGTs tumors, and was most frequently found in SACCs (47.3%, 26/55) (Figure 2A, 2C, Figure S1 and Table 1). Among the SACC patients, loss of PTEN expression were predominantly found in the poorly differentiated, high grade malignancy, i.e. the solid SACCs (81.8%, 18/22), and in 11.8% (2/17) tubular and 37.5% (6/16) cribriform SACCs (Figure 2B, 2C and Table 1). The average of PTEN staining index is 6.76 in tubular, 3.94 in cribriform, and 1 in the solid SACCs (Table 1).

**Table 1:** The correlation between clinicopathological data and PTEN expression in salivary gland tumors

Factor	No.	Average PTEN index	PTEN IHC expression		
			Normal	Reduced	Loss
SGTs	114	5.55	43 (37.7%)	44 (38.6%)	27 (23.7%)
SACCs Tubular Cribriform Solid	55 17 16 22	3.64 6.76 3.94 1	12(21.8%) 9 (52.9%) 3 (18.7%) 0 (0%)	17 (30.9%) 6 (35.3%) 7(43.8%) 4 (18.2%)	26 (47.3%) 2 (11.8%) 6 (37.5%) 18 (81.8%)^*^
PAs MECs BCCs MyECs AICs	24 20 5 5 5	6.5 7.25 8.4 9 9	8 (33.3%) 9 (45%) 4 (80%) 5(100%) 5 (100%)	15 (62.5%) 11 (55%) 1 (20%) 0 (0%) 0 (0%)	1 (4.2%) 0 (0%) 0 (0%) 0 (0%) 0 (0%)
NSG	20	9	20 (100%)	0 (0%)	0 (0%)
Age					
< 60	73	5.51	26 (35.6%)	31 (42.5%)	16 (21.9%)
≥ 60	41	5.63	17 (41.5%)	13 (31.7%)	11 (26.8%)
Gender					
Male	47	5.91	18 (38.3%)	20 (42.6%)	9 (19.1%)
Female	67	5.30	25 (37.3%)	24 (35.8%)	18 (26.9%)
Tumor site					
Major SGs	80	5.31	29 (36.3%)	36 (44.9%)	15 (18.8%)
Minor SGs	34	4.94	14 (41.1%)	8 (23.6%)	12 (35.3%)

* *p* < 0.05 (chi-square test)

We have recently reported that the solid pattern SACC is one of the poor prognostic factors in SACC patients [12]. The higher incidence of PTEN loss in solid pattern SACC prompts us to analyze the patients' survival situation and correlate with their PTEN expression. Kaplan-Meier analysis was performed in 53 SACC patients, and showed a significant correlation between patients' survival and PTEN expression level (Figure S2).

The average PTEN staining index in PAs was 6.5, and about 4.2% (1/24) PAs lost PTEN expression (Figure 2A, Figure S3 and Table 1). The average PTEN staining index were 7.25 in MECs, and 8.4 in BCCs and about 55% of MECs and 20% of BCCs exhibited reduced PTEN expression (defined as PTEN staining index between 3 to 6) (Figure 2A, Figure S3 and Table 1). Last, PTEN was well expressed in either MyECs or AICs, and there were no loss or reduced PTEN expression in both SGTs (Figure 2A, Figure S3 and Table 1).

### Reduced PTEN expression activated S6 ribosome protein and correlated with HER2 overexpression in human SACCs

The higher incidence of PTEN negative cases in human SACCs prompted us to further investigate the role of PTEN in tumorigenesis of SACCs. We first examined the activation status of two downstream genes of PI3K pathway, phosphorylation of AKT (pAKT) and S6 ribosome protein (pS6) in human SACC samples. While most of the tubular or cribriform SACCs retained PTEN expression, these cases exhibited undetectable or low level of pAKT or pS6 (Figure 2C). In contrast, most solid SACCs lost PTEN expression and almost all these cases showed high level of pS6 staining (94.4%, 17/18). In contrast, only 27.8% (5/18) PTEN-negative cases showed AKT activation (Figure 2C and 2E). We further examined several molecules which have been reported to be involved in human SACCs [9, 13, 14]. The percentages of positive pS6 and HER2 cases were significantly higher than other three molecules, i.e pAKT, MYB and EGFR. (Figure 2D and 2E).

### Reduced expression of PTEN in human SACC cell lines correlated with migration and invasion *in vitro* and tumor size *in vivo*

**Figure 3:**
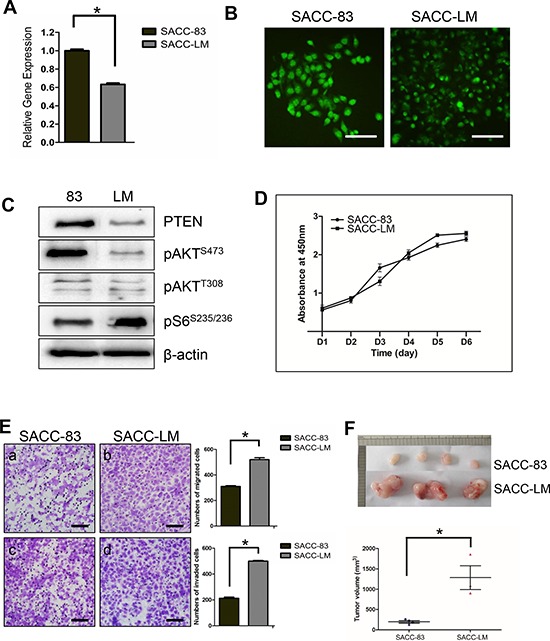
Reduced expression of PTEN in human SACC cell lines correlated with migration, invasion *in vitro* and tumor size *in vivo* **A.** qPCR quantitation of PTEN mRNA level in SACC-83 and SACC-LM cell lines. *p < 0.05 **B.** IF staining of PTEN in SACC-83 and SACC-LM cell lines. The scale bars represent 100 μm **C.** Western blotting of PTEN, pAKT and pS6 in SACC-83 (83) and SACC-LM (LM) cell lines. β-actin was used as a loading control. **D.** Cell proliferation assay on SACC-83 and SACC-LM cell lines using CCK-8 method. **E.** Migration assay (a–b) and invasion assay (c–d) on SACC-83 and SACC-LM cell lines. The results are quantified in the right panels. *p < 0.05. **F.** *In vivo* xenografts of SACC-83 and SACC-LM cell lines in athymic mice. 4 mice were injected SACC-83 cell line, and 3 mice were injected SACC-LM cell line. Mice tumors were harvested at day 30 after injection. The quantitation of tumor volume is shown below. *p < 0.05.

We then utilized a pair of human SACC cell lines SACC-83 and its metastasis derivative SACC-LM to investigate the role of PTEN in SACC development and progression. Reduced level of endogenous PTEN expression was detected in the SACC-LM cell line compared to the SACC-83 cell line at both mRNA level as examined by qRT-PCR (Figure 3A), and protein level as examined by either IF (Figure 3B), or Western blotting (Figure 3C). Interestingly, similar to the IHC results from human SACC tissues, reduced PTEN level in the SACCC-LM cells correlated with S6 activation, but not with AKT activation at both phosphorylation sites (Figure 3C). Cell proliferation assay, however, didn't show significance difference between SACC-83 and SACC-LM (Figure 3D); Instead, SACC-LM cells exhibited more migratory and invasive abilities compared to the SACC-83 cells (Figure 3E). We further subcutaneously injected both SACC cell lines into nude mice. Tumors started to grow at day 12 for both SACC-83 and SACC-LM, and were harvested at day 30. As shown in Figure 3F, SACC-LM cell line formed significantly larger size of tumor compared to SACC-83 cell line.

### Knocking down PTEN increased cell proliferation, migration and invasion *in vitro*, and tumor size *in vivo*

**Figure 4:**
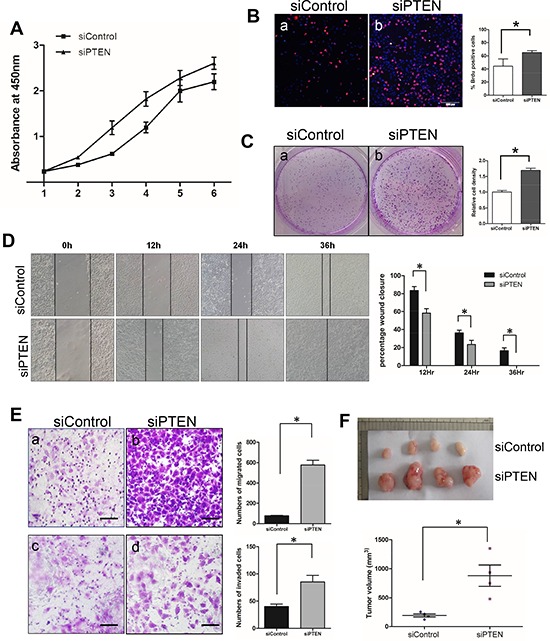
Knocking down PTEN increased cell proliferation, migration and invasion *in vitro*, and enhances tumor size *in vivo* **A.** Cell proliferation assay by CCK-8 method on SACC-83 cell lines transfected with either control siRNA, pSURE (siControl) or PTEN siRNA, pSURE/siPTEN (siPTEN). *p < 0.05. **B.** BrdU incorporation assay on SACC-83 cell lines transfected with either control or PTEN siRNA. The result is quantified in the right panel. The scale bar represents 200 μm. *p < 0.05. **C.** Clonogenic assay on SACC-83 cell lines transfected with either control or PTEN siRNA. The result is quantified in the right panel. *p < 0.05. **D.** Scratch wound assay on SACC-83 cell lines transfected with either control or PTEN siRNA. The result is quantified in the right panel. *p < 0.05 **E.** Migration assay (a–b) and invasion assay (c–d) on SACC-83 cell lines transfected with either control (a, c) or PTEN siRNA (b, d). The results are quantified in the right panels. *p < 0.05 **F.** *In vivo* xenografts of SACC-83 cell lines transfected with either control (upper) or PTEN siRNA (lower). The quantitation of tumor volume is shown in the lower panel. *p < 0.05.

To further assess the role of PTEN in SACC tumorigenesis, we transfected PTEN siRNA into the SACC-83 cell line. Nearly 80% PTEN knockingdown was achieved as examined by Western blotting (Figure S4A). Correspondingly, both phosphorylation sites of AKT and one phosphorylation site of its downstream signaling effector pS6 are activated upon PTEN silencing, suggested a solid link between PTEN and the PI3K/AKT pathway (Figure S4B). Cell proliferation assay showed significantly increase of cell proliferation in siPTEN cells compared to siControl cells (Figure 4A). Increased cell proliferation was further confirmed by higher percentage of BrdU positive cells (Figure 4B), and more colonies (Figure 4C) in the siPTEN cells. We then used scratch wound assay and migration/invasion chamber assay to assess the role of PTEN in cell migration and invasion. PTEN knockingdown significantly accelerated wound closure as early as 12 hours after wounding (Figure 4D). Increased cell migration and invasion were further elucidated in siPTEN cells by the migration (Figure 4Ea, b) and invasion (Figure 4Ec, d) chamber assays. Lastly, we injected SACC83-siControl and SACC83-siPTEN cells into nude mice. Tumor started to grow after 9 days after injection of SACC83-siPTEN cells compared to 12 days after injection of SACC83-siControl cells. By the 30 days when we harvested tumors, the average tumor volumes of SACC83-siPTEN cells were about 4.5 times bigger than those of SACC83-siControl cells (Figure 4F). Taken together, both *in vitro* and *in vivo* data clearly demonstrated a tumor suppressor role of PTEN in SACC tumorigenesis.

To further understand the underlying molecular mechanisms of PTEN loss in SACC tumorigenesis, we have performed a gene expression array on SACC-83 cells transfected with siPTEN/siControls. We particularly examined genes related to PI3K/mTOR pathway which are directly related to the PTEN silencing and cell proliferation. Although the majorities of molecules in PI3K/mTOR pathway are not significantly affected at mRNA level, one interesting molecule, Unc-51 like autophagy activating kinase 1(ULK1), is significantly downregulated upon PTEN silencing (Figure S5A). ULK1 has been identified as one the key molecules for regulating cell autophagy [15]. The strong correlation between PTEN and ULK1 warrants a further study of PTEN in autophagy and cancer. In addition, downregulation of E-cadherin encoding gene CDH1 and upregulation of vimentin (Figure S5B) suggests the role of PTEN in cell migration and epithelial-mesenchymal transition.

### Treatment with a dual PI3K and mTOR inhibitor, NVP-BEZ235 decreased cell proliferation, migration and invasion

**Figure 5:**
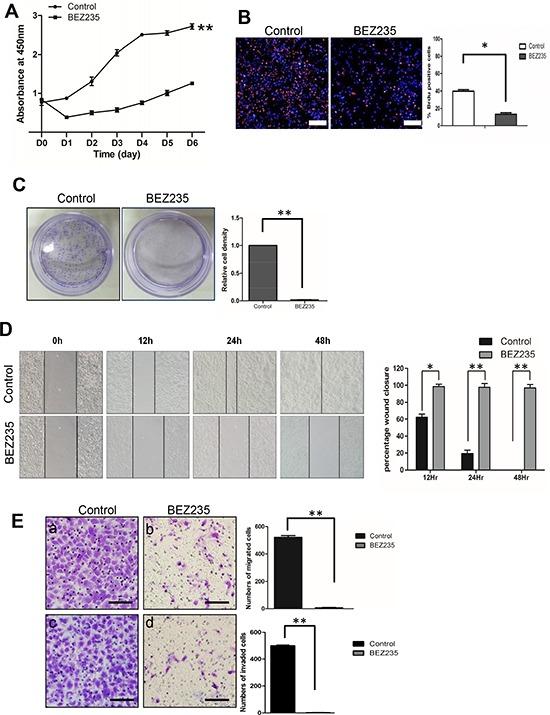
Treatment with a dual PI3K and mTOR inhibitor, NVP-BEZ235, decreased cell proliferation, migration and invasion **A.** Cell proliferation assay by CCK-8 method on SACC-LM cell lines treated with either DMSO (control) or NVP-BEZ235 (BEZ235). **p < 0.01 **B.** BrdU incorporation assay on SACC-LM cell lines treated with either DMSO (control) or NVP-BEZ235 (BEZ235). The result is quantified in the right panel. The scale bar represents 200 μm. *p < 0.05 **C.** Clonogenic assay on SACC-LM cell lines treated with either DMSO (control) or NVP-BEZ235 (BEZ235). The result is quantified in the right panel. **p < 0.01 **D.** Scratch wound assay on SACC-LM cell lines treated with either DMSO (control) or NVP-BEZ235 (BEZ235). The result is quantified in the right panel. *p < 0.05, **p < 0.01 **E.** Migration (a, b) and Invasion (c, d) assays on SACC-LM cell lines treated with either DMSO (control) or NVP-BEZ235 (BEZ235). The result is quantified in the right panels. The scale bars represent 100 μm **p < 0.01.

The correlation between PTEN loss and increased phosphorylation of S6 ribosome protein indicated activation of the PI3K/mTOR pathway, providing an opportunity to test if a pharmaceutical approach will be effective to control SACC tumorigenesis. We first treated SACC-83 cells and SACC-LM cells with a dual PI3K/mTOR inhibitor, NVP-BEZ235. We observed a significant reduction of phosphorylation of AKT and S6 as early as 4 hours treatment (Figure S6). Correspondingly, cell proliferation was significantly inhibited upon NVP-BEZ235 treatment in SACC83-LM cells (Figure 5A) and SACC-83 cells (Figure S7A). The treatment also significantly reduced BrdU positive cells (Figure 5B), and almost diminished cell colonies in both cells (Figure 5C, and Figure S7B). Furthermore, NVP-BEZ235 treatment decreased cell migration in both cells as assessed by scratch wound assay (Figure 5D, and Figure S7C), and migration chamber assay (Figure 5Ea, b), and cell invasion as determined by invasion chamber assay (Figure 5Ec, d, and Figure S7D).

### Treatment with NVP-BEZ235 reduced tumor burden and lung metastasis in a SACC xenograft mouse model

To further evaluate the efficacy of NVP-BEZ235 inhibitor to treat SACC *in vivo*, we subcutaneously injected SACC-LM cells into nude mice to establish a SACC xenograft mouse model. Histological examination revealed several histological similarities of these xenografts to human SACCs. Tumor cells arranged densely in small duct-like (Figure S8A, A'), small clumps (Figure S8B, B') and solid cord-like (Figure S8C, C') structure. The small duct-like structure made form 2–3 layers of cells scattered throughout the tumor and sometimes the cavity containing the mucus. Dedifferentiated tumors have irregular tumor islands composed of anaplastic cells with abundant cytoplasm and desmoplastic stroma (Figure S8D, D'), and some tumor cells are filled with some mucus-like material in certain area (Figure S8E, E'). IHC staining of myoepithelial markers of p63 (Figure S9A, A'), α-SMA (Figure S9B, B'), and calponin (Figure S9C, C') and basal cell markers of K5 (Figure 9D, D') and K8/18 (Figure 9E, E') further showed the histological similarities between human SACCs and tumors from the SACC cell lines.

**Figure 6:**
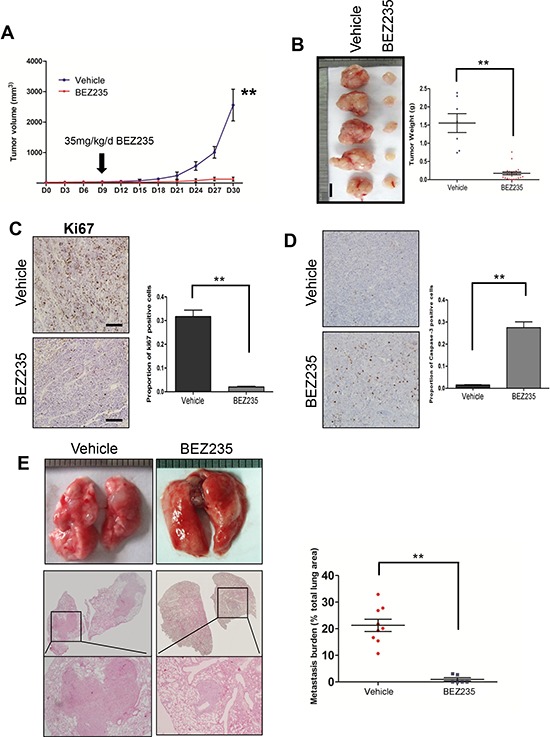
Treatment with a dual PI3K/and mTOR inhibitor reduced tumor burden and lung metastasis **A.** Schematics of tumor kinetics and treatment plan using either NVP-BEZ235 (BEZ235) or vehicle control (vehicle) on SACC-LM xenograft. **B.** Representative images from SACC-LM xenograft tumor treated with 21 days of either vehicle or NVP-BEZ235. The tumor weight is summarized in the right panel. The scale bar represents 1 cm. **C.** IHC of cell proliferation marker Ki67 in the SACC-LM xenografts treated with 21 days of either vehicle or NVP-BEZ235. The result is quantified in the right panel. **p < 0.01 **D.** IHC of cell apoptosis marker caspase 3 in the SACC-LM xenografts treated with 21 days of either vehicle or NVP-BEZ235. The result is quantified in the right panel. **p < 0.01 **E.** HE slides showing treatment effect for lung metastasis using NVP-BEZ235 on the experimental metastasis by tail injection of SACC-LM cells. The percentage of lung metastasis area is quantified in the right panel. **p < 0.01 for A–E.

We then applied 35 mg/kg NVP-BEZ235 po. at day 7 after injection of SACC-LM cells, and treated once a day for a consecutive 21 days. This treatment was well tolerated in mice and didn't generate obvious adverse effects during the treatment. Tumor started to grow beginning at day 15 in the vehicle control group and at day 24 in the NVP-BEZ235 treated group (Figure 6A). We harvested tumors at day 30, and observed significantly smaller tumors upon NVP-BEZ235 treatment. The average tumor weight was nearly 90% reduced (0.17 g vs. 1.55 g) in the NVP-BEZ treatment group compared to the control group (Figure 6B). NVP-BEZ235 treatment also inhibited cell proliferation *in vivo* as examined by Ki67 IHC staining (2.05% vs. 31.7%, Figure 6C), and induced cell apoptosis *in vivo* as shown by the percentage of caspase 3 positive cells (27.5% vs 1.5%, Figure 6D). Correspondingly, both phosphorylation sites of AKT, one phosphorylation of pS6 were significantly diminished upon NVP-BEZ235 treatment. The activity of the AKT-downstream pro-apoptotic protein BAD was also reduced after the treatment (Figure 10S).

We then assessed the therapeutical efficacy of NVP-BEZ235 on lung metastasis. We intravenously injected SACC-LM cells, applied 35 mg/kg NVP-BEZ235 po. daily for 21 days, and harvested mice 21 days after injection. Histological examination showed that 9 out of 9 mice developed lung metastasis in the control group, while 2 out of 6 mice developed lung metastasis in the NVP-BEZ235 treatment group. NVP-BEZ235 treatment also significantly reduced the size and numbers of lung metastases (Figure 6E). These *in vivo* data showed a promising therapeutic efficacy of using NVP-BEZ235 to treat both SACC tumors and their lung metastases.

## DISCUSSION

In this study, we examined PTEN expression in both normal human salivary glands and a variety of human SGTs, including two most common types SACC and MEC. We also compared PTEN expression with several epithelial (luminal) and myoepithelial/basal (abluminal) cell markers by IHC staining. We found that PTEN was expressed in all luminal and abluminal cells of intercalated, striated, and excretory ducts of normal human salivary gland. PTEN was also expressed in abluminal acini cells, but not in luminal acini cells. This ubiquitous expression pattern of PTEN suggests a common role of PTEN in suppressing a variety of human SGT development. Indeed, we found about 23.7% SGT cases with loss of PTEN expression in a total of 114 human SGTs, which is consistent with a recently report (21.6%) [9]. However, we found most of the SGT cases with loss of PTEN expression were human SACCs, while Etti et al reported a higher cases of loss of PTEN expression in salivary duct carcinomas and squamous cell carcinomas [9].

SACC is the second most common SGTs in humans. It is characterized by endless growth, diffuse invasion, a high rate of local recurrence and metastasis, causing a substantial mortality. Based on the components of its histological cell types, SACC is roughly divided into three major patterns, i.e. cribriform, tubular, and solid pattern. Among the three, solid pattern is the most poorly differentiated, high grade malignancy with higher rate of recurrence and metastasis. We have shown solid pattern of SACC has the lowest survival rate in our more than ten years patient's follow-up [12]. Interestingly, our study here found that over 80% of solid SACC lost PTEN expression, significantly higher than other two patterns of SACCs, highlighting the important role of PTEN in suppressing development of solid SACC, and loss of PTEN in solid SACC progression. In addition, using the Kaplan-Meier survival analysis, we found the correlation between PTEN expression and SACC patinets' survival. Supporting our finding, the association between PTEN and disease prognosis and lymph node metastasis in human SGTs has also been currently reported [9, 16].

The molecular mechanisms for PTEN loss in human SGTs are still not fully understood. A germline mutation has been reported in human epithelial myoepithelial cacinomas of salivary gland [17], and a case of acinic cell carcinoma patient [11], respectively. However, two recent studies using whole exome sequencing revealed that somatic mutations of individual gene are generally low, except the common MYB-NFIB gene fusion in human SACCs. There is only one SACC case of PTEN mutation in a total of 80 SACC cases they analyzed [18, 19]. In contrast, either homozygous or hemizygous deletion of PTEN has been reported in over 20% of human SGTs and correlated with PTEN expression [9]. The results of PTEN methylation in human SGTs are controversial and inconclusive [20, 21]. In addition to these genetic or epigenetic changes, posttranslational modification or certain microRNAs targeting PTEN are also possible to be responsible for the reduced expression of PTEN in human SGTs. Further investigation is warranted, which may provide novel therapeutical opportunities for these patients.

Loss of PTEN expression usually results in activation of AKT/mTOR pathway due to its antagonizing function to the PI3K/AKT/mTOR pathway, and activation of PI3K/AKT/mTOR pathway has been reported in human SGTs [22]. This correlation was also evidenced in the SACC-83 cells transfected with siPTEN. The solid correlation between PTEN and PI3K/mTOR pathway provides an excellent opportunity of using inhibitors targeting PI3K/mTOR for targeted therapy for human SGTs. However, only less than 30% of cases of loss of PTEN expression exhibited AKT activation, while nearly 95% cases showed S6 activation in our SGT patients, suggesting in some cases of loss of PTEN expression, activation of mTOR will not be always dependent on AKT status. Similar results of uncoupling between PI3K/mTOR and AKT activation have been observed [23, 24]. We also examined three molecular alterations commonly seen in human SGTs [25]. Both HER2 and MYB, but not EGFR overexpression correlated with the loss of PTEN expression cases. Both targeting HER2 and EGFR as novel therapy for human SGTs have been proposed and tested [1, 2, 26]. Our data suggest that targeting mTOR, or HER2, instead of AKT, or EGFR may be more effective in human SACC treatment.

Followed our study on human clinical SGT samples, we also functional evaluated the role of PTEN in human SACC cell lines. Reduced PTEN expression was observed in the lung metastasis counterpart SACC-LM compared to the primary SACC line SACC-83, suggesting loss of PTEN may also suppress lung metastasis of SACC. Interestingly, increased S6 activation, but not AKT were observed in the SACC-LM cell lines compared to the SACC-83 cell line, suggesting activation of mTOR, but not AKT may dominantly drive SACC progression. Further experiment of PTEN knocking down in the SACC83 cell line significantly increased cell proliferation, colony forming, cell migration and invasion *in vitro*, and increased tumor size *in vivo*. Interestingly, a recent report of using genetically engineered mouse model of PTEN and APC developed spontaneous SGT [27]. These data strongly support that PTEN is a potent tumor suppressor in human SGT development and progression.

Besides the well-documented role of PTEN in the PI3K/mTOR pathway, we are also interested to explore the novel function of PTEN in SACC tumorigenesis. To this point, our microarray data suggested two interesting pathways, cellular autophagy and epithelial mesenchymal transition are possible affected by the PTEN expression. This pilot result warrants a detailed mechanistic investigation of PTEN in human SACC tumorigenesis in future.

Current therapeutic options for human SGT are still very limited [1–3, 26]. Thus, the development of novel, effective therapies to treat patients with SGTs is critical for improving current therapeutic protocol. In our study, almost all the SGT patients with reduced PTEN expression manifested activation of PI3K/mTOR. By using a PI3K/mTOR inhibitor NVP-BEZ235, strong growth inhibition were observed in both SACC-83 and SACC-LM cell lines. NVP-BEZ235 treatment also strongly reduced cell migration and invasion. More importantly, a potent treatment efficacy on primary tumor size and distant lung metastasis upon NVP-BEZ235 treatment was observed in the SGT xenograft mouse model. Correspondingly, the activities of AKT and pS6 as well as one of the pro-apoptotic protein BAD in the PI3K/AKT pathway were significantly diminished upon the NVP-BEZ235 treatment. This finding strongly supports the rationale of using PI3K/mTOR inhibitor for SGT patients, particularly on patients with loss of PTEN expression. Similar treatment efficacy was also reported in the PTEN genetically engineered mouse model in that the mTOR inhibitor rapamycin treatment led to complete regression of SGT tumor growth [27]. Furthermore, partially response but no toxic effect of using mTOR inhibitor, Temsirolimus on two salivary gland cancers patients with cutaneous metastasis was reported recently [28], providing a strong rationale of applying mTOR inhibitor for SGT treatment. In our study, we also found reduced PTEN expression was correlated with HER2 overexpression. This result is in consistent with the recent report that deletion of PTEN correlated with HER2 gene amplification. However, in contrast to their findings, we didn't observe the correlation between PTEN deletion and EGFR overexpression [9]. While this result may be further supportive of using HER2 inhibitor, it also raise question of using EGFR inhibitor in the current targeted therapy for SGT patients. Interestingly, no significant clinical response of a phase II study of gefibinib in advanced SGT patients was recently reported [29].

In summary, our study showed a significantly reduction of PTEN in human SGTs, particularly in the solid pattern of human SACCs. This reduction of PTEN correlated with activation of PI3K/mTOR and HER2 overexpression, but not with AKT activation or EGFR overexpression. We further functionally validated a potent tumor suppressing role of PTEN in human SGT development and progression. Lastly, experimental therapy of using PI3K/mTOR inhibitor NVP-BEZ235 successfully reduced primary tumor growth and lung metastasis. Our results highlighted the importance of using PI3K/mTOR inhibitor in the treatment for human SGT patients with reduced PTEN expression.

## MATERIALS AND METHODS

### Patients and clinical samples

All patient samples were collected from the Dalian Medical University and the 9th People's Hospital of Shanghai Jiao Tong University during 2001 to 2013 under the approvals of the protocol by both Institutional Review Boards. Diagnosis of the SGTs in this study was evaluated by head and neck cancer pathologists and informed consent was obtained from each patient. The samples comprised of a total of 114 SGTs, including 55 salivary adenoid cystic carcinomas (SACCs), 24 pleomorphic adenomas (PAs), 20 mucoepidermoid carcinomas (MECs), and 5 cases of each basal cell carcinomas (BCCs), myoepithelial carcinomas (MyECs), and acinic cell carcinomas (AICs). 20 normal salivary gland (NSG) tissues were also included in this study. The demographic and clinic-pathological information for each patient is listed in Table 1.

### Immunohistochemistry (IHC)

Formalin-fixed, paraffin-embedded slides from either human tissues, or mouse xenografts were used for the following IHC analysis according the protocol we have described [30]. Antibody binding was visualized by the VECTORSTAIN ABC kit (Vector Labs, Burlingame, CA, USA, PK-4001) and DAB Kit (Maixin Biotech, Fujian, China, DAB-1031), and counterstained with haematoxylin. Pictures were taken by the Olympus cellSens software, and a BX43 microscope (OLYMPUS) for light microscopy. The primary antibodies for human tissues used were PTEN (Zymed, San Francisco, CA, USA; 18–0256), phospho-AKT-Ser473 (Cell signaling, Beverly, MA, USA, #9271), phospho-AKT-Thr308 (Cell signaling, Beverly, MA, USA, #4056), phospho-S6 ribosomal protein (Cell signaling, Beverly, MA, USA, #2211), EGFR (Zymed, San Francisco, CA, USA, 28–0005), v-Myb+c-Myb (Abcam, Cambridge, UK, ab45150), and HER2 (Thermo Scientific, San Jose, CA, MS325B0). The primary antibodies for the mouse xenografts were Ki67 (Maixin Biotech, Fujian, China, MAB-0672), Calponin (Maixin Biotech, Fujian, China, MAB0335), cytokeratin K8/18 (Maixin Biotech, Fujian, China, MAB0650), p63 (Maixin Biotech, Fujian, China, MAB0365), cytokeratin 5 (Thermo Scientific, San Jose, CA, MS-1896), S100 (Maixin Biotech, Fujian, China, Kit-0007–2) and caspase 3 (Cell signaling, Beverly, MA, USA, #9664).

### Immunofluorescence (IF)

Double IF was carried on the formalin-fixed, paraffin-embedded slides from human normal salivary tissues to examine PTEN (Zymed, San Francisco, CA, USA, 1:100, 18–0256) distribution compared with α-SMA (Beyotime Institute of Biotechnology, Jiangsu, China, 1:500, AA132), p63 (Maixin Biotech, Fujian China, ready-to-use, MAB0365), and Cytokeratin 5 (Thermo Scientific, San Jose, CA, 1:200, MS-1896) using the protocol we described previously [30]. Double IF labeling was achieved by mixing Alexa Fluor488-conjugated goat anti-rabbit IgG and Fluor594-conjugated goat anti-mouse IgG (Molecular Probes, Invitrogen, Carlsbad, CA, 1:500). Nuclei were labeled with 4, 6′-diamidino-2-phenylindole (DAPI, Beyotime Biotech, Jiangsu, China, 1:500). Images were collected with a BX43 fluorescence microscope (OLYMPUS) using cellSens software.

### Quantitative analysis of immunostainings

The staining intensity was graded using a four-point scale: 0, no staining; 1, light yellow; 2, brown; and 3, dark brown (Figure S1). The proportion of cells stained was assessed using a four-point scale: 0, < 10% staining; 1, 11–50% staining; 2, 51–75% staining; and 3, > 75% staining. The staining index (intensity multiplies proportion) was determined as follows: 0–2, loss of staining, 3–6, reduced staining, and 7–9, normal staining.

### Cell culture

SACC-83 cell line was established from a patient with SACC in the sublingual gland. SACC-LM cell line was derived from the parental SACC-83 through *in vivo* selection from the lung metastatic foci, and has a higher lung metastasis rate compared to SACC-83 [31, 32]. Both cell lines were kindly provided by Dr. Sheng-Lin Li of Peking University School and Hospital of Stomatology, China. Cells were cultured in RPMI 1640 medium (GIBCO, Carlsbad, CA, USA) containing 10% fetal bovine serum (FBS, GIBCO, Carlsbad, CA, USA) at 37°C in 5% CO_2_ cell incubator (Thermo Scientific).

### Transfection

PTEN knockdown was achieved by transfected PTEN siRNA (pSURE/siPTEN, Qiagen, Valencia, CA, USA). The SACC-83 cells were seeded at 5 × 10^4^ cells/well in 6-well plate, and transfected with pSURE/siPTEN PTEN siRNA using Lipofectamine™ 2000 Transfection Reagent (Invitrogen, Carlsbad, CA, USA), according to the manufacturer's protocol. After 24 hours, cells with transient transfection PTEN siRNA were harvested for the subsequent experiments.

### RNA extraction and quantitative PCR

Total RNA was isolated from cells by TRIzol Reagent (Takara, Japan), according to the manufacturer's instruction. cDNA was synthesized from total RNA using PrimeScript™ RT reagent Kit with gDNA Eraser (Perfect Real Time, Takara, Japan, DRR047). Quantitative PCR was performed by using SYBR Premix Ex TaqTM II kit (Takara, Japan, DRR0820) running on the TP800 Thermal Cycler Dice Real Time System (Takara). GAPDH served as an endogenous control gene. The PTEN primer was used: human PTEN forward 5′-GAGCGTGCAGATAATGACAAGGAAT-3′ and reverse 5′-GGATTTGACGGCTCCTCTACTGTTT-3′. All the qPCR experiments were performed in triplicates, and the results were analyzed using 2^−ΔΔCt^ method as we described recently [30].

### Western blot analysis

Protein was isolated in ice-cold RIPA buffer containing complete protease/phosphatase inhibitors (KeyGEN Biotech, Nanjing, China, KGP250), and quantified by BCA method. 60 μg of total protein were underwent SDS-PAGE and transferred to a nitrocellulose membranes. The membrane were then blocked in the 5% skim milk solution in TBST and the incubated with the primary antibody at 4°C overnight, and subsequently incubated with the HRP-conjugated secondary antibody at room temperature for 2 hours. Signals were detected using ECL Western blotting substrate (Thermo Scientific, Ashville, NC). Antibodies directed against PTEN (#9559), phospho-AKT-Thr308 (#4056), phospho-AKT-Ser473 (#9271), phospho-S6 Ribosomal Protein (1:500, #2211) were all purchased from Cell Signaling Technology (Cell signaling, Beverly, MA, USA).

### Cell proliferation

For cell proliferation assay, SACC cell lines were seeded in 96-well plates (Costar) at 5000 cells per well. Cell Counting Kit-8 (CCK-8) assay (Dojindo, Kyushu, Japan) was used to assess cell proliferation rate at each time point according to the manufactory's protocol. Absorbance values were measured at the wavelength of 450 nm. For the clonogenic assay, a total of 5000 cells were plated in 6 cm plates. The plates were incubated for 8 days and stained with 0.1% (w/v) crystal violet. Colonies were counted under a microscope. For BrdU incorporation assay, 5-bromo-2′-deoxyuridine (BrdU) (Sigma-Aldrich, St. Louis, MO, B5002) at a final concentration of 10 μM was added to the culture medium for 24 hours. Subsequently, cells were fixed and IF was done with mouse anti-BrdU antibody (Sigma-Aldrich, St. Louis, MO, 1:200, B8434).

### Migration and invasion

Cell migration was assessed by scratch wound assay. Monolayer cells were grown until confluence and a scratch wound with 400–600 μm distance was introduced with a 200 μl pipette tip. Wound closure was measured under the microscope for 36 to 48 hours.

Transwell assays were performed using Transwell chambers (Corning Costar, #3422) with a polycarbonate membrane (6.5 mm diameter, 8 μm pore size) with/without Matrigel (BD Biosciences, 354480). Cells were counted in five individual fields on each insert and calculated by Image-Pro Plus Version 7.0 Software.

### Xenograft models

All animal experiments were approved the IACUC committee of Dalian Medical University. A total of forty-six 6-to 8-wk-old male athymic nude mice were used. Tumorigenicity was assessed after the subcutaneously injection of 5 × 10^6^ cells; metastasis was evaluated after the i.v. injection of 2 × 10^6^ cells. Tumors volumes were recorded every 3 days and calculated with the formula V = (Length × Width^2^)/2. Tumor and lung tissues were harvested and fixed in 4% PFA for histological characterization.

### Inhibitor treatment

For *in vitro* experiments, cells were seeded in T75 flasks until becoming ~80% confluent. A final concentration of 500 nM NVP-BEZ235 (Novartis, Basel, Switzerland) dissolved in DMSO was then added for 4 hours. For *in vivo* experiments, we injected either 5 × 10^6^ SACC-LM cells subcutaneously or 2 × 10^6^ SACC-LM cells intravenously at day 0. Nine days after injection, mice were randomized into controls and treated groups, and received 35 mg/kg/day dissolved in 10%NMP/90%PEG300 (sigma-aldrich, St. Louis, MO, #328634, and #90878) daily for 21 days by oral gavage. The control group was orally treated with the vehicle only. Mice were sacrificed after 21 days of treatment and the tumor and lung tissues were harvested.

### Gene expression microarray

Total RNAs of each group were used for generation of Cy3-labeled complementary RNA (cRNA) for the Agilent Whole Human Genome Oligo Microarray (4 × 44K) (Agilent Technologies, Santa Clara, CA, USA), containing 41,000 human transcripts. Ratio was calculated by comparison between siPTEN group and siControl group. Genes were analyzed and the heatmap was generated using the SBC analysis system (Shanghai Biotechnology Corporation, Shanghai, China).

### Statistical analysis

Data results for individual assays represent the means ± SEM. All calculations were done using IBM SPSS Statistics 19. Statistical comparisons were made using the two-tailed Student's *t*-test. All *p*-values of less than 0.05 were considered statistically significant. GraphPad Prism 5.0 software was used to create graphs.

## SUPPLEMENTARY FIGURES


