# Supplementary material for: High frequency of loss of PTEN expression in human solid salivary adenoid cystic carcinoma and its implication for targeted therapy
Source: Oncotarget. 2015 Mar 20;6(13):11477–91. doi: 10.18632/oncotarget.3411 (PMC4484470; doi:10.18632/oncotarget.3411)
Supplement: Supplementary file 1 [file oncotarget-06-11477-s001.pdf]

SUPPLEMENTARY FIGURES

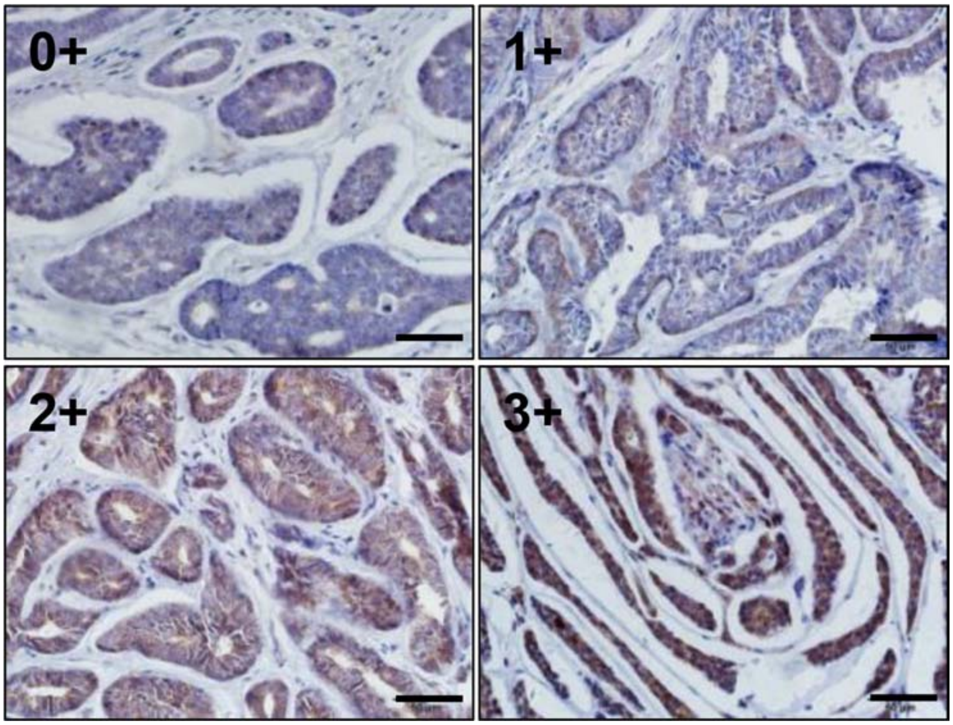

Supplementary Figure S1: Representative images of staining intensity score for PTEN immunohistochemistry. The scale bars represent 50 μm.

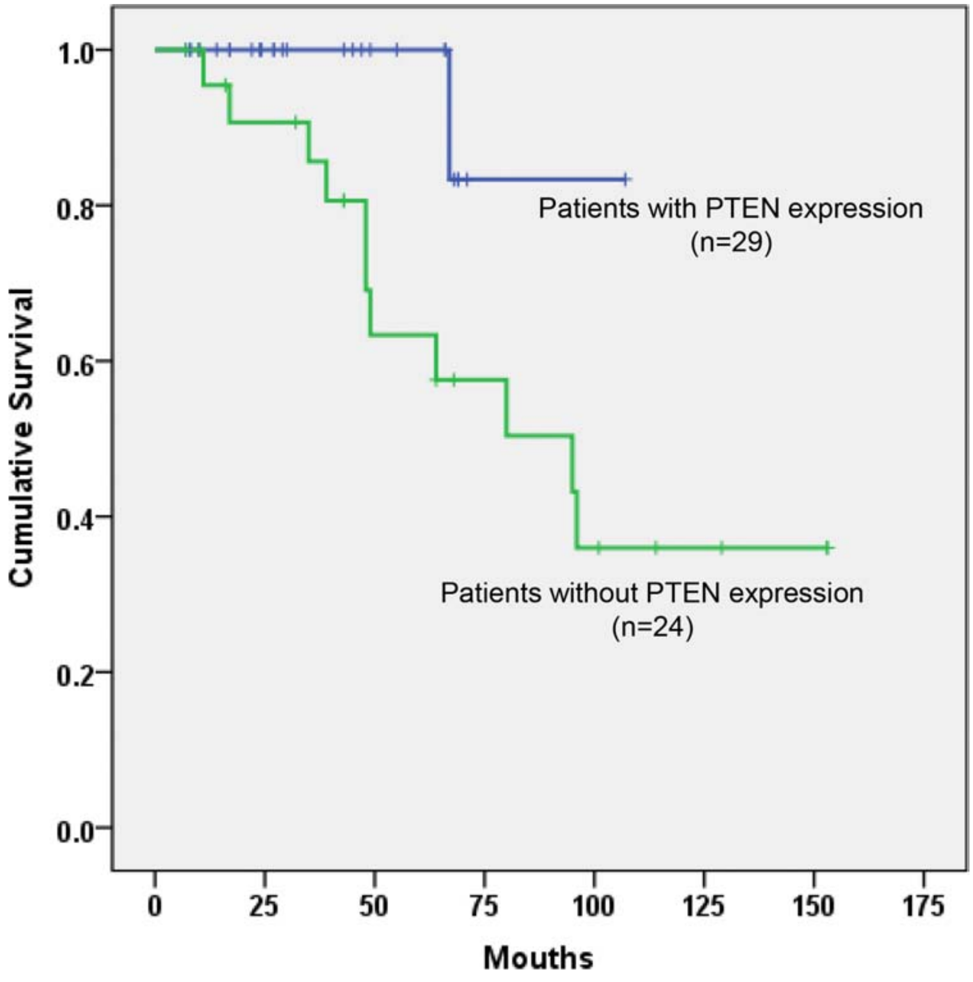

Supplementary Figure S2: Kaplan-Meier survival curve of the 53 SACC patients with PTEN expression (*n* = 29, blue line) and without PTEN expression (*n* = 24, green line). *p* value equal to 0.03.

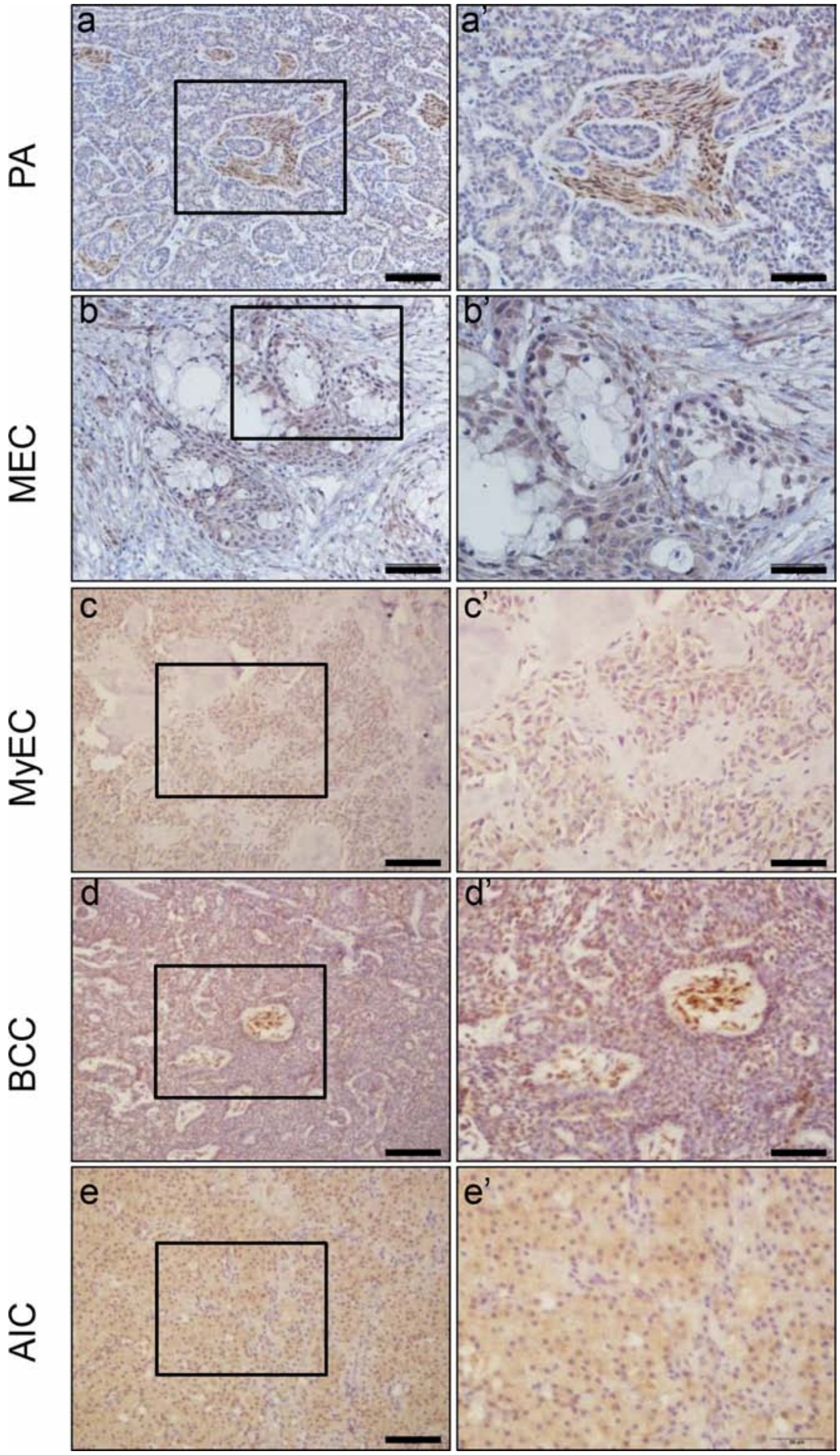

Supplementary Figure S3: Representative images of PTEN IHC staining in human pleomorphic adenoma (PA: a, a'), mucoepidermoid carcinoma (MEC: b, b'), myoepithelial carcinoma (MyEC: c, c'), basal cell carcinoma (BCC: d, d'), and acinic cell carcinoma (AIC: e, e'). The scale bars represent 100 µm in **a-e.** and 50 µm in **a'-e'.**

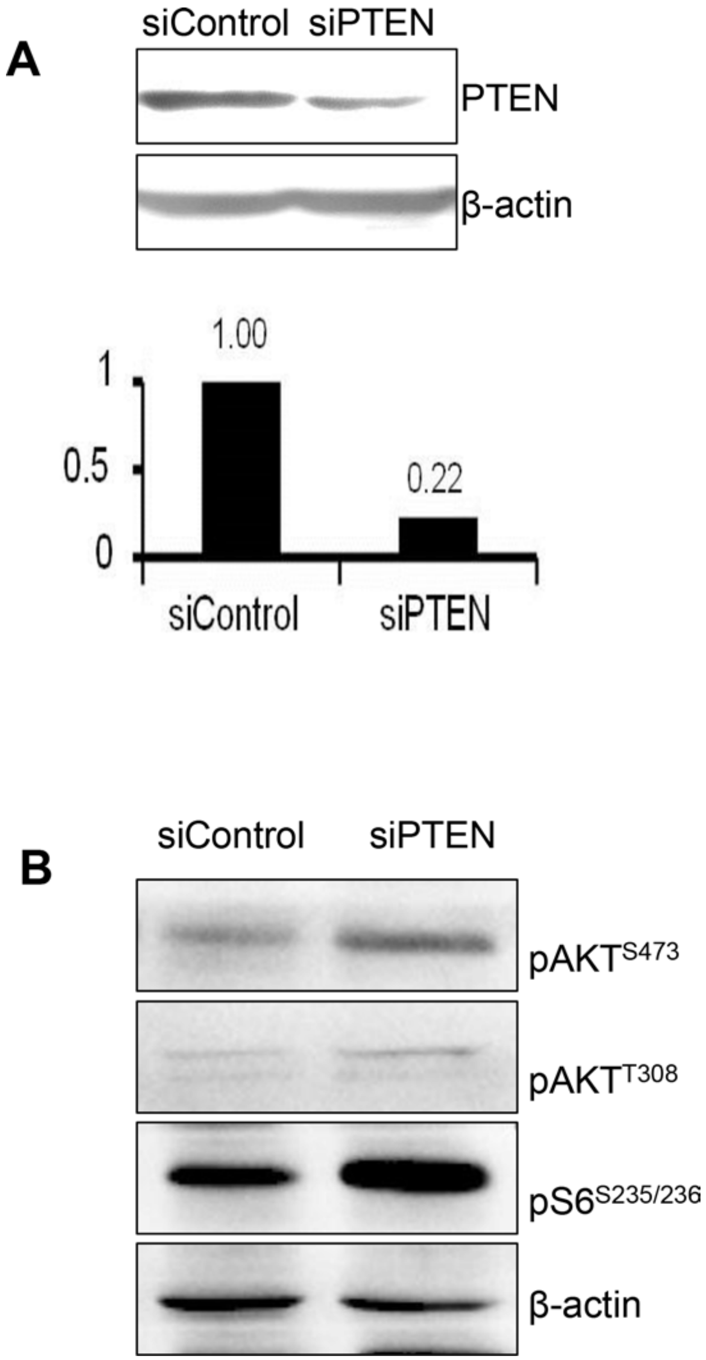

Supplementary Figure S4: A. Western blotting showed the PTEN knocking down level in the SACC-83 cells transfected with either pSURE (control) or pSURE/siPTEN plasmids. The result is quantified in the lower panel after normalization with internal control β-actin. **B.** Western blotting on SACC-83 cell line transfected with PTEN siRNA.

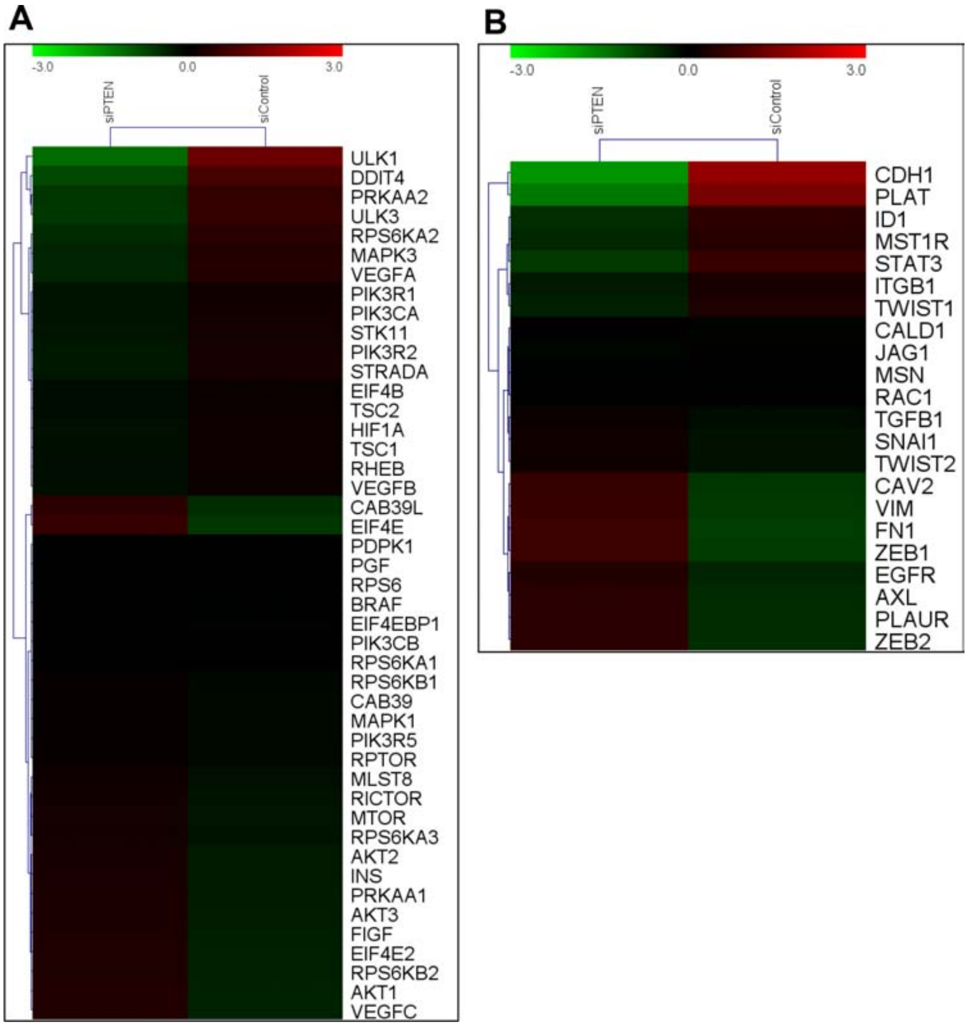

Supplementary Figure S5: Heatmap of gene expression array showing the differential expression of genes related to PI3K/mTOR pathway A. and migration and invasion B.

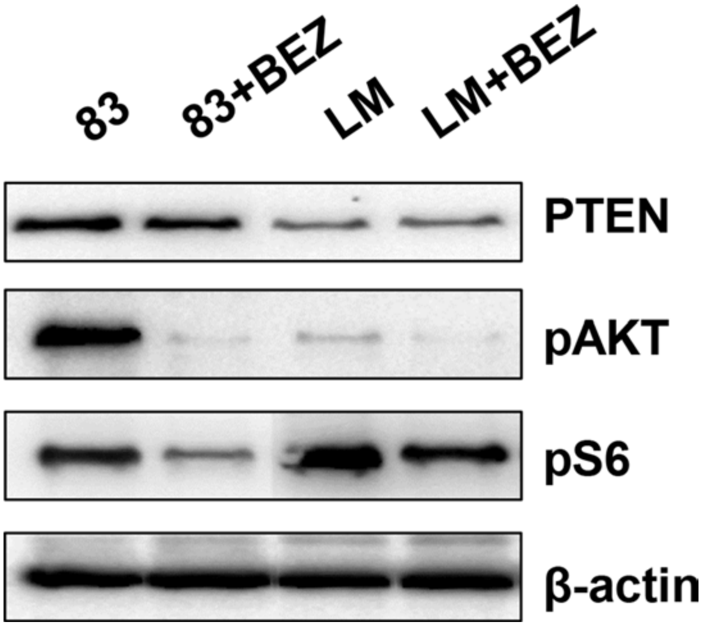

Supplementary Figure S6: Western blotting on SACC-83 (83) and SACC-LM (LM) cell lines treated with a dual PI3K and mTOR inhibitor NVP-BEZ235 (BEZ).

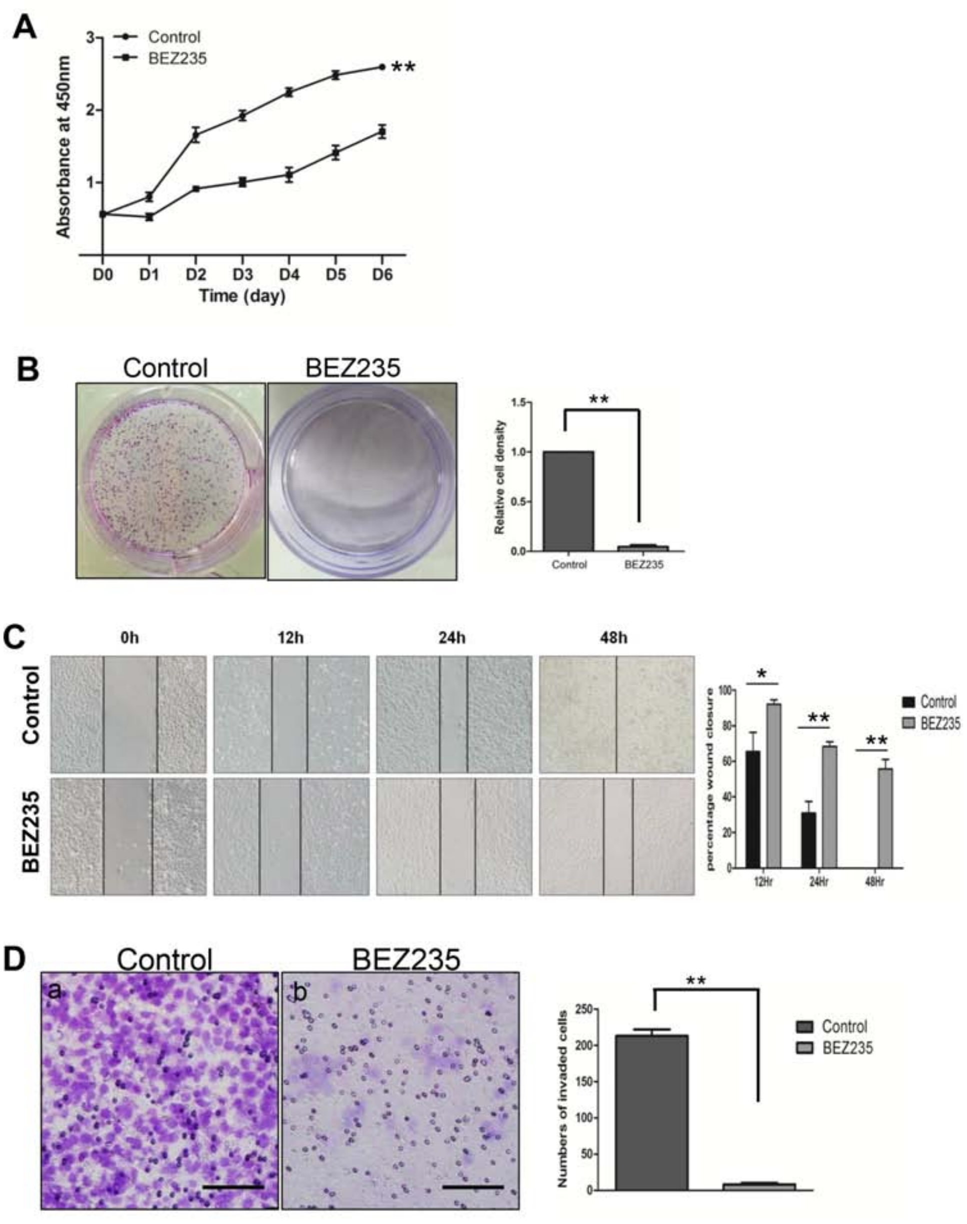

Supplementary Figure S7: Treatment with a dual PI3K and mTOR inhibitor on the SACC-83 cell line decreased cell proliferation, migration and invasion. A. Cell proliferation assay by CCK-8 method on the SACC-83 cell line treated with either DMSO (control) or NVP-BEZ235 (BEZ235). $^{**}p < 0.01$ **B.** Clonogenic assay on the SACC-83 cell line treated with either DMSO (control) or NVP-BEZ235 (BEZ235). The result is quantified in the lower panel. $^{**}p < 0.01$ **C.** Wound scratch assay on the SACC-83 cell line treated with either DMSO (control) or NVP-BEZ235 (BEZ235). The result is quantified in the right panel.$^{*}p < 0.05$, $^{**}p < 0.01$ **D.** Invasion assay on the SACC-83 cell line treated with either DMSO (control) or NVP-BEZ235 (BEZ235). The result is quantified in the right panel. $^{**}p < 0.01$

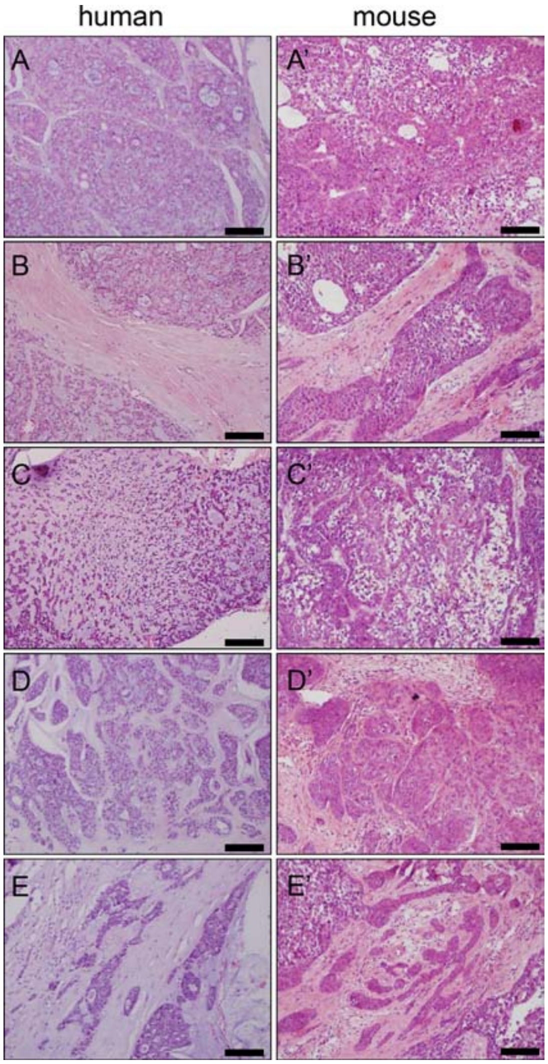

Supplementary Figure S8: Histological similarities between human SACCs A-E. and mouse SACC-LM xenografts A'-E'. HE sections showed the similarities of small duct-like structure (A, A'); desmoplastic stroma (B, B'), mucus-like material (C, C'), solid cord-like structure (D, D') and small clumps structure (E, E'). Scale bar, 100 µm.

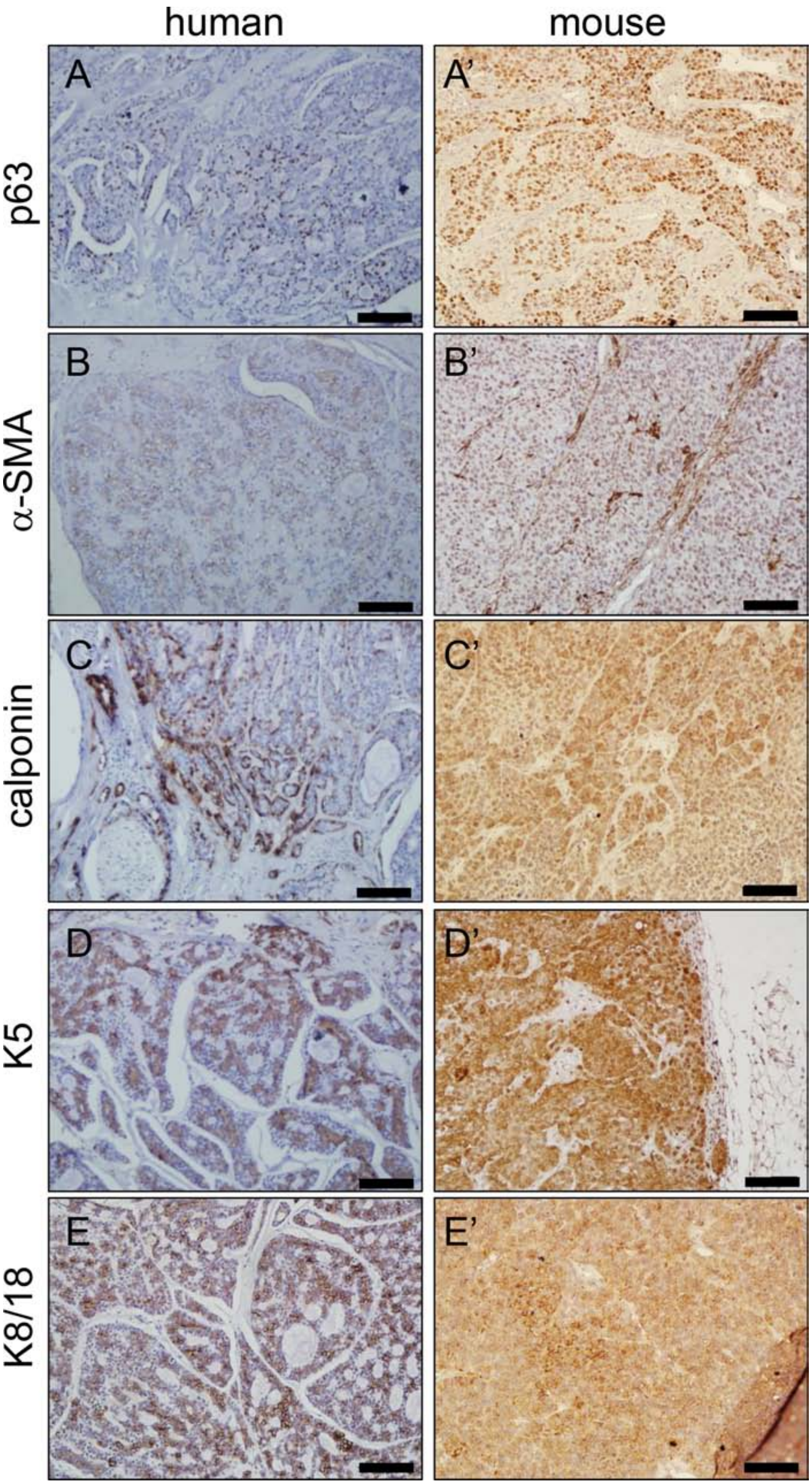

Supplementary Figure S9: Molecular similarities of SACC markers between human SACCs A-E. and mouse SACC-LM xenografts A'-E'. IHC showed the similarities of expression of myoepithelial markers, p63 (A, A'), α-SMA (B, B') and Calponin (C, C'), basal marker K5 (D, D') and glandular epithelial marker CK8/18 (E, E'). Scale bar, 100 μm.

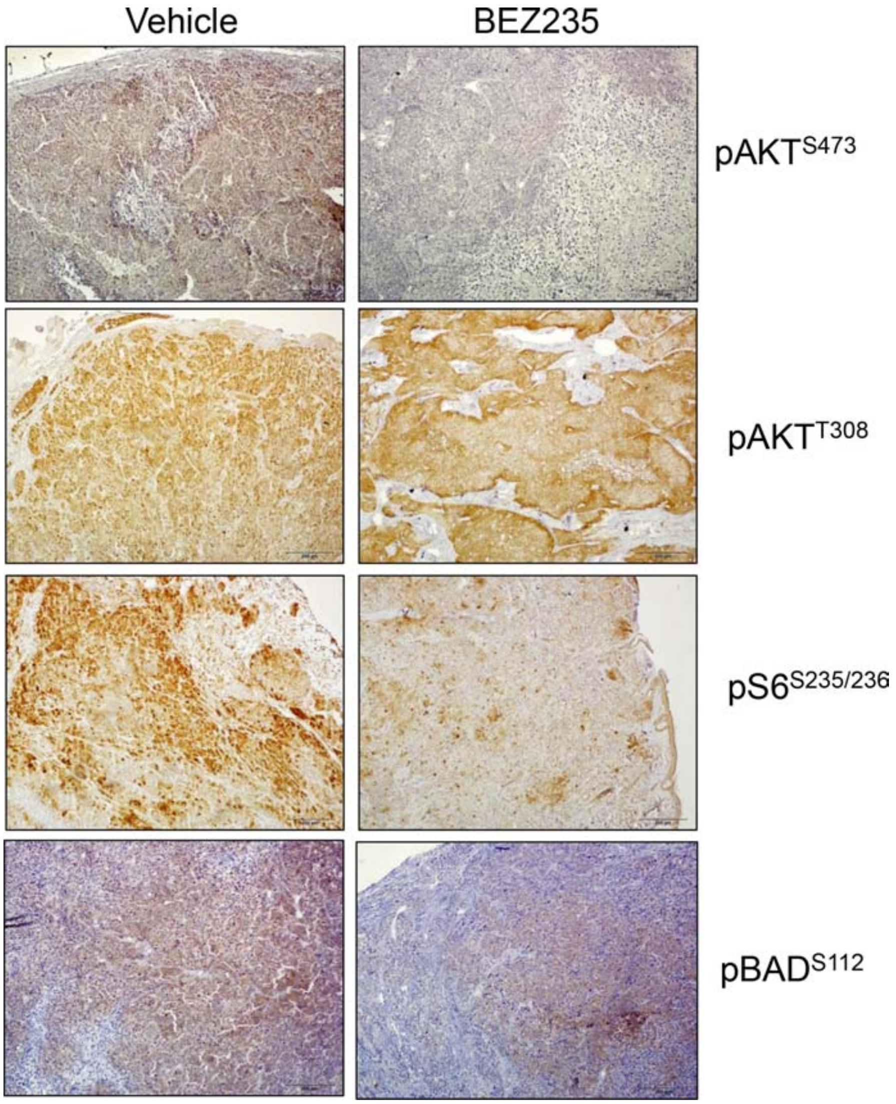

Supplementary Figure S10: Representative images of IHC staining of pAKTS473, pAKTT308, pS6S235/236 and pBADS112 in the SACC-LM xenografts treated with either NVP-BEZ235 or the vehicle control.
